# Effect of Iodine treatments on *Ocimum basilicum* L.: Biofortification, phenolics production and essential oil composition

**DOI:** 10.1371/journal.pone.0226559

**Published:** 2019-12-16

**Authors:** Claudia Kiferle, Roberta Ascrizzi, Marco Martinelli, Silvia Gonzali, Lorenzo Mariotti, Laura Pistelli, Guido Flamini, Pierdomenico Perata

**Affiliations:** 1 Department of Pharmacy, University of Pisa, Pisa, Italy; 2 PlantLab, Institute of Life Sciences, Scuola Superiore Sant’Anna, Pisa, Italy; 3 Department of Agriculture, Food and Environment, University of Pisa, Pisa, Italy; Institute for Biological Research, SERBIA

## Abstract

Iodine biofortification has been gaining interest in recent years as a sustainable and innovative approach to eradicate iodine deficiency disorders. Studying the impact of iodine biofortification on plant phenotype, biochemical and physiological parameters is crucial to leverage the expertise and best practices for the agro-food industry and human health. The aim of this study was to evaluate iodine biofortification on the main quantitative and qualitative traits of basil (*Ocimum basilicum* L.) plants cultivated both in open field and in growth chamber. The impact of KI and KIO_3_ treatments was evaluated on biomass production, as well as on the synthesis of phenolic compounds, especially rosmarinic acid and other caffeic acid derivatives, and on the essential oil (EO) composition. These compounds are typically accumulated in basil leaves and strongly contribute to the plant nutraceutical value and aroma. In open field, the use of increasing concentrations of both iodine salts gradually enhanced iodine accumulation in leaves, also determining an increase of the antioxidant power, total phenolics, rosmarinic acid and cinnamic acid accumulation. The composition of EO was only slightly affected by the treatments, as all the samples were characterized by a linalool chemotype and a minor alteration in their relative content was observed. A growth chamber experiment was performed to test EO variation in controlled conditions, broadening the range of iodine concentrations. In this case, plant chemotype was significantly affected by the treatments and large EO variability was observed, suggesting that iodine form and concentration can potentially influence the EO composition but that in open field this effect is overcome by environmental factors.

## Introduction

It is well known that iodine is an essential mineral for human health, thus an inadequate intake of this element can lead to different diseases called “Iodine deficiency disorders” such as goiter, cretinism or hyper- and hypothyroidism [1]. World Health Organization recommends an iodine dietary intake of 150 μg/day for adult men, 250 μg/day for pregnant women [2] and a value between 90–120 μg/day for children [3]. However, an excess of iodine intake can be harmful and the American Thyroid Association established at 1100 μg/day the maximum threshold for iodine intakes to avoid toxicity effects and thyroid dysfunctions [4].

Iodine is available for humans through diet. Nevertheless, apart from seaweeds, fish and shellfish, which are good sources of iodine, foods usually contain low concentrations of the element, far from meeting the recommended daily intakes. In particular fruit and vegetables, which are at the base of the food pyramid, are generally poor in iodine [5]. The use of iodized salt represents the main strategy adopted to integrate diet with iodine. However, in recent years several attempts have been experimented to directly enrich vegetable foods. To increase the iodine content in crops, different “biofortification” strategies can be adopted, such as agronomic practices, transgenic techniques or conventional plant breeding [6].

Iodine biofortification through the administration of iodine salts, generally KI or KIO_3_, has been widely experimented in different plant groups such as leafy vegetables (i.e. spinach, lettuce, cabbage), horticultural fruit crops (i.e. tomato, eggplant, pepper), fruit trees (i.e. plum, nectarine), tubers (i.e. potato and carrot), and staple crops (i.e. wheat, rice, maize) [7], thanks to the fact that soluble iodine forms can be readily taken up from soil by roots or even absorbed by leaves. Physiological and molecular processes at the base of iodine uptake and accumulation are far from being fully characterized. On the basis of current knowledge, iodine can be absorbed by roots through aspecific carriers or channels, even if the presence of specific transporters cannot be excluded [7] and it can enter leaf cells via stomata and/or cuticular waxes [8]. Nevertheless, the accumulation of iodine in the plant has not to be detrimental for the final yield. Several papers indeed demonstrated that the use of high concentrations of iodine can be toxic for plants, reducing both growth and biomass. The main processes associated with iodine phytotoxicity are the reduction of CO_2_ assimilation, due to the decrease in leaf size, stomatal conductance and the photosynthetic pigment content [9–11], and the negative influence/antagonistic interaction of iodine with mineral nutrient uptake [12–14], even if contrasting evidences on iodine-induced nutrient deficiency are present in literature [11,15], suggesting a minor role of this mechanism on iodine toxicity. On the other hand, applications of very low amounts of iodine can be beneficial, promoting the plant growth and inducing the production of phenolic compounds which are positively involved in the plant response to biotic and abiotic stresses [16]. In particular, working with lettuce, Blasco et al. [17] demonstrated that the application of low KIO_3_ concentration (<80 μM) enhanced the plant resistance to salt stress, thanks to the increase in hydroxycinnamic acids production, associated with phenylalanine ammonia-lyase (PAL) activation.

Among phenolic derivatives regulated by PAL activity in the shikimate pathways, phenylpropenes and other aromatic compounds are important components of the plant aroma [18], which is one of the most important ecological traits, being involved in protection against pests, pathogens and predators and in attraction of pollinators [18,19].

The yield and composition in terms of bioactive volatile compounds, such as phenolics and essential oils (EOs) depends on genetic, environmental and agronomic factors [20], and strongly influences the nutraceutical and commercial value of the crop. In particular, the quality of aromatic/medicinal plants deeply relies on their EO composition, which is responsible for their flavor and scent. Considering the possible influence of iodine on the production of secondary metabolites, the impact of iodine on flavor and scent volatiles should be evaluated, too.

*Ocimum basilicum* L. is an herbaceous and annual plant typically cultivated in Mediterranean areas and known as common basil. The plant belongs to *Lamiaceae* and, as much as other species of this family, it is mainly used for culinary or medicinal purposes because of its high concentration of antioxidant phenolic compounds, such as rosmarinic acid and other caffeic acid derivatives (CADs) [21,22] and due to the typical aroma derived from its EOs. The distinction between the numerous basil varieties is largely based on their EO composition, which is of the utmost importance in consumers’ preference. The large consumption of basil as a food ingredient makes it a possible candidate for biofortification purposes. Moreover, iodine is predominantly translocated in plants through the xilematic flux [7], thus making leafy vegetables, such as basil, the preferred crops to be enriched with iodine. However, considering the possible use of basil as an aromatic and medicinal herb, its nutraceutical value also relies on the complex mix of secondary metabolites, which are synthesized in the leaves, and the possible interference of iodine with them has to be carefully examined.

In the present study, an iodine biofortification protocol was developed for basil plants cultivated both in open field and growth chamber. The main purpose of the work was to verify the possibility to enrich the iodine basil content at levels suitable for human consumption and to evaluate the impact of KI or KIO_3_ treatments on the production of secondary metabolites of interest, such as phenolic compounds including rosmarinic acid and selected CADs, and EOs.

To the best of our knowledge, no papers have been published so far on the influence of iodine on EO composition in plants.

## Materials and methods

### Plant material

Basil (*Ocimum basilicum* L.) cv. Superbo was used in two separate experiments conducted in open field or growth chamber.

### Growing conditions and experimental set-up

#### Open field experiment

A field experiment was carried out in 2018 in a farm located in Sarzana (Italy, 44°03'19.4"N 9°59'55.4"E). The land accessed was privately owned; the permit and approval obtained for the work were provided by Marco Nicolini, the legal representative and owner of the “Società Agricola Nicolini Marco” farm. As scheduled in the commercial protocol adopted, basil plants were transplanted approx. in mid—May and, during the cultivation, they were mowed every 10–12 days, until the end of the commercial season. The plant density was about 160,000 plants ha^-1^ and the spacing between rows and plants was 25 and 15 cm, respectively. Basil was cultivated on a sandy-loam soil (10.9% heavy clay, 66.6% sand and 22.5% loam), characterized as follows: organic matter 2.8%, cation exchange capacity 16.2 (meq/100g), pH_H2O_ 8.0, EC 0.19 mS cm^-1^, N 1.8 g kg^-1^, P 22.0 mg kg^-1^, K 78.2 mg kg^-1^, Mg 175.2 mg kg^-1^, Ca 1256.0 mg kg^-1^.

Irrigation was provided daily by aspersion and after every mowing 166.6 kg ha^-1^ (13.5 N-46,2 K_2_O-38,4 K) and 83.3 kg ha^-1^ (34.2 N: 17.3% N-NO_3_, 16,9% N-NH_4_) of commercial fertilizers were applied, by adding them to irrigation water.

The iodine content quantified in soil and in the nutrient solution averaged between 4.13 ± 0.53 mg kg^-1^ and 21 ± 1 μg L^-1^, respectively.

At the beginning of August, after the commercial mowing, the irrigation of the experimental area was stopped and after two days plants were watered with a solution of KI or KIO_3_ (Sigma Aldrich, St. Louis, MO, USA) at different concentrations (0; 0.1; 1.0; 10 mM). Treatments were applied twice, every two days, each plant receiving approx. 80 ml of solution/treatment. The experiment was arranged according to a randomized complete block design with three replications, each plot measuring 1.8 m x 3 m (5.4 m^2^).

Sampling was performed after two days from the second treatment by removing the whole aerial part of the plants from the soil. Leaves were immediately frozen in liquid nitrogen and stored at -80°C for biochemical analysis or used for EO determinations. The whole plants dry weight (DW) was also determined by desiccating them in a ventilated oven (70°C) until constant weight (16 plants from each plot/replication).

#### Growth chamber experiment

Basil seeds were individually sown in 5.5 ø cm pots filled with peat-based commercial substrate (product name: Aussaaterde, Hawita Vechta,Germany) and vernalized for two days at 4°C. Main characteristics of the used substrate were: organic matter 40%, cation exchange capacity 105 (meq/100g), pH_H2O_ 6.1, EC 0.6 mS cm^-1^, N 80 mg kg^-1^, P 80 mg kg^-1^, K 90 mg kg^-1^. Plants were cultivated in a growth chamber at 22°C day—18°C night with 12-h photoperiod and quantum irradiance of 100 μmol photons m^-2^ sec^-1^. During the growing cycle, plants were fertirrigated every three days with a nutritive solution containing the following concentrations of macro and micronutrients: 1.25 mol m^-3^ KNO_3_, 1.50 mol m^-3^ Ca(NO_3_)_2_, 0.75 mol m^-3^ MgSO_4_, 0.50 mol m^-3^ KH_2_PO_4_, 50 mmol m^-3^ KCl, 50 mmol m^-3^ H_3_BO_3_, 10 mmol m^-3^ MnSO_4_, 2.0 mmol m^-3^ ZnSO_4_, 1.5 mmol m^-3^ CuSO_4_, 0.075 mmol m^-3^ (NH_4_)Mo_7_O_24_, 72 mmol m^-3^ Fe-EDTA. Salts were dissolved in tap water and the absence of iodine in the nutrient solution was verified (<1 μg L^-1^, threshold value of inductively coupled plasma-mass spectrometry—ICP-MS). Electrical conductivity (EC) and pH were respectively 0.6 dS m^-1^ and 6.0. The soil iodine content averaged 1.99 ± 0.99 mg kg^-1^. After one month of cultivation, the fertirrigation was stopped and after two days plants were watered with a solution of KI or KIO_3_ at different concentrations (0; 10 μM; 0.1 mM; 1 mM; 10 mM; 100 mM), following the same schedule used in the field experiment: treatments were applied twice, every two days, each plant receiving approx. 30 ml of solution/treatment. Sampling was performed two days after the last treatment and the fresh leaves were analyzed for the iodine content and the EO composition. The plant DW was also determined as previously reported (12 plants for each treatment).

### Phytochemical analysis

#### Iodine content

The iodine content was determined in leaf samples by ICP-MS (Perkin Elmer, Optima 7300 DV) after tetramethylammonium hydroxide (TMAH) extraction at 70°C [23,24]. Iodine was also quantified in soil and in the basal nutrient solution used. Three biological replicates were analyzed.

#### Chlorophyll and carotenoid content

The total content of chlorophylls and carotenoids was determined spectrophotometrically and expressed on a fresh weight (FW) basis. Apical leaf samples (20 mg) were extracted with 2 ml methanol (v/v) overnight at 4°C in the dark, under continuous agitation. The extracts were centrifuged (5 min at 5.000 rpm) and subsequently analyzed at 665.2, 654.6 (chlorophylls) and 470 nm (carotenoids). Chlorophyll and carotenoid content was calculated according to Lichtenthaler [25]. Four biological replicates were analyzed.

#### Phenolic content and antioxidant capacity

The two assays were performed on the same methanolic extract obtained as follows: fresh leaf samples (0.5 g) were homogenized with 5 ml of 70% methanol (v/v) and extracted overnight at 4°C in the dark, under continuous agitation. After centrifugation (5 min at 5.000 rpm) the clear supernatant was collected and used for determinations. Four biological replicates were analyzed.

#### Total phenolics

The determination was performed spectrophotometrically using the colorimetric Folin-Ciocalteu assay. Briefly, 125 μl of methanolic extract were mixed with 0.5 ml of deionized water and 125 μl of the Folin-Ciocalteu reagent (Sigma Aldrich). After 5 min of incubation at room temperature (RT), 1.25 ml of a 7% sodium carbonate solution and 1 ml of deionized water were added and the solution was left to stand at RT for 90 min. Absorbance was read at 765 nm and the total amount of phenolics was expressed in terms of gallic acid equivalents, using gallic acid as standard. Four biological replicates were analyzed.

#### Antioxidant capacity

The antioxidant capacity was evaluated by using the DPPH test, which is considered one of the simplest, fastest and less expensive method for this kind of determination [26]. The solution of DPPH (1,1-Diphenyl-2-picrylhydrazyl, Sigma Aldrich) in 70% (v/v) methanol was prepared, resulting in the final concentration of DPPH being 0.25 mM. Different volumes of leaf methanolic extract (from 2 to 10 μl) were added to 335 μl of DPPH solution, to reach the final volume of 1 ml. The mixture was vortexed and left to stand at RT for 30 min in the dark. A blank solution (control) was prepared by mixing 70% methanol with DPPH radical solution.

Absorbance was read spectrophotometrically at 517 nm. The DPPH radical scavenging percentage (%) was calculated using the formula [(A_0_-A_1_)/A_0_] × 100, where A_0_ is the absorbance of the control, and A_1_ is the absorbance of the sample. The leaf antioxidant capacity was expressed as inhibitory concentration (IC_50_), which is the sample concentration required to scavenge 50% of the initial DPPH concentration, calculated by plotting inhibition percentage against extract concentration. Four biological replicates were analyzed.

#### Caffeic acid derivatives (CADs) determination

Approximately 500 mg of fresh leaves were extracted as described by Kiferle et al., 2011 [25]. Subsequently, methanol was evaporated under vacuum at 35°C and the aqueous phase was partitioned against ethyl acetate, after adjusting the pH to 2.8. Samples were dried and silylated with N,O-bis (trimethylsilyl) trifluoroacetamide containing 1% trimethylchlorosilane (Pierce, Rockford, IL, USA) at 70°C for 1 h. Chromatography-tandem mass spectrometry (GC-MS/MS) analysis was performed on a Saturn 2200 quadrupole ion trap mass spectrometer coupled to a CP-3800 gas chromatograph (Varian Analytical Instruments, Walnut Creek, CA, USA) equipped with a MEGA 1MS capillary column (30 m × 0.25 mm i.d., 0.25 μm film thickness) (Mega, Milano, Italy). The carrier gas was helium, which was dried and air free, with a linear speed of 60 cm s^-1^. The oven temperature was maintained at 80°C for 2 min and increased to 300°C at a rate of 10°C min^-1^. Injector and transfer line were set at 250°C and the ion source temperature at 200°C. Full scan mass spectra were obtained in EI+ mode with an emission current of 10 μA and an axial modulation of 4 V. Data acquisition was from 150 to 600 Da at a speed of 1.4 scan s^-1^. Final data are the means of three biological replicates.

The analytes quantification was performed using the calibration curve of the following standards: chlorogenic acid, t-cinnamic acid, p-coumaric acid, ferulic acid, caffeic acid (Sigma Aldrich) and rosmarinic acid (Extrasynthese S.A., Genay, France). The minimum level of quantification and the minimum level of detection were monitored daily with standard and with the signal/noise ratio, respectively.

### Essential oils extraction and analyses

Fresh leaves were roughly cut from plants prior to extraction. All the EOs were hydrodistilled with a standard Clevenger apparatus for 2 hours. The hydrodistilled EOs were diluted to 0.5% in HPLC-grade *n*-hexane and then injected into a GC–MS apparatus. Gas chromatography–electron impact mass spectrometry (GC–EIMS) analyses were performed with an Agilent 7890B gas chromatograph (Agilent Technologies Inc., Santa Clara, CA, USA) equipped with an Agilent HP-5MS (Agilent Technologies Inc.) capillary column (30 m × 0.25 mm; coating thickness 0.25 μm) and an Agilent 5977B single quadrupole mass detector (Agilent Technologies Inc.). Analytical conditions were as follows: injector and transfer line temperatures 220 and 240°C, respectively; oven temperature programmed from 60 to 240°C at 3°C min^-1^; carrier gas helium at 1 ml min^-1^; injection of 1 μl (0.5% HPLC grade *n*-hexane solution); split ratio 1:25. The acquisition parameters were as follows: full scan; scan range: 30–300 m/z; scan time: 1.0 sec. Identification of the constituents was based on a comparison of the retention times with those of the authentic samples, comparing their linear retention indices relative to the series of n-hydrocarbons. Computer matching was also used against commercial (NIST 14 and ADAMS) and laboratory-developed mass spectra library built up from pure substances and components of known oils and MS literature data [27–32].

### Statistics

Experimental data on plant DW and phytochemical analyses were analyzed by one way ANOVA using the Statgraphics Plus 5.1 program (StatPoint, Inc., Herdon, VA, USA). Means values were separated according to the Tukey’s test, at P≤0.05.

Multivariate statistical analyses on EOs were carried out with the JMP Pro 13.2.1 software package (SAS Institute, Cary, NC, USA). For all the EOs composition, both the hierarchical cluster analysis (HCA) and the principal component analysis (PCA) were performed. Both the HCA and the PCA methods can, indeed, be applied to observe groups of samples even when there are no reference samples that can be used as a training set to establish the model. The HCA was performed by the Ward’s method on unscaled data. The PCA was achieved selecting the two highest principal components (PCs) obtained by the linear regressions operated on mean-centered, unscaled data. As an unsupervised method, this analysis aimed at reducing the dimensionality of the multivariate data of the matrix, whilst preserving most of the variance [33]. For the statistical evaluation of the complete EOs composition of both the open field and growth chamber samples, the covariance data was a 88 x 18 matrix (88 individual compounds x 18 samples = 1584 data). The chosen PC1 and PC2 cover 79.00% and 14.84% of the variance, respectively, for a total explained variance of 93.84%.

## Results and discussion

### Open field experiment

The goal of the field experiment was the evaluation of the effect of iodine on basil production from a quantitative and qualitative point of view. After the addition of different concentrations of IO^−^_3_ or I^−^ (0; 0.1; 1.0; 10 mM) as potassium salts to the irrigation water, the biomass production was determined and leaves were characterized for the iodine content and several biochemical parameters such as chlorophylls, carotenoids, phenol compounds, CADs, antioxidant power and EO composition.

**Fig 1:**
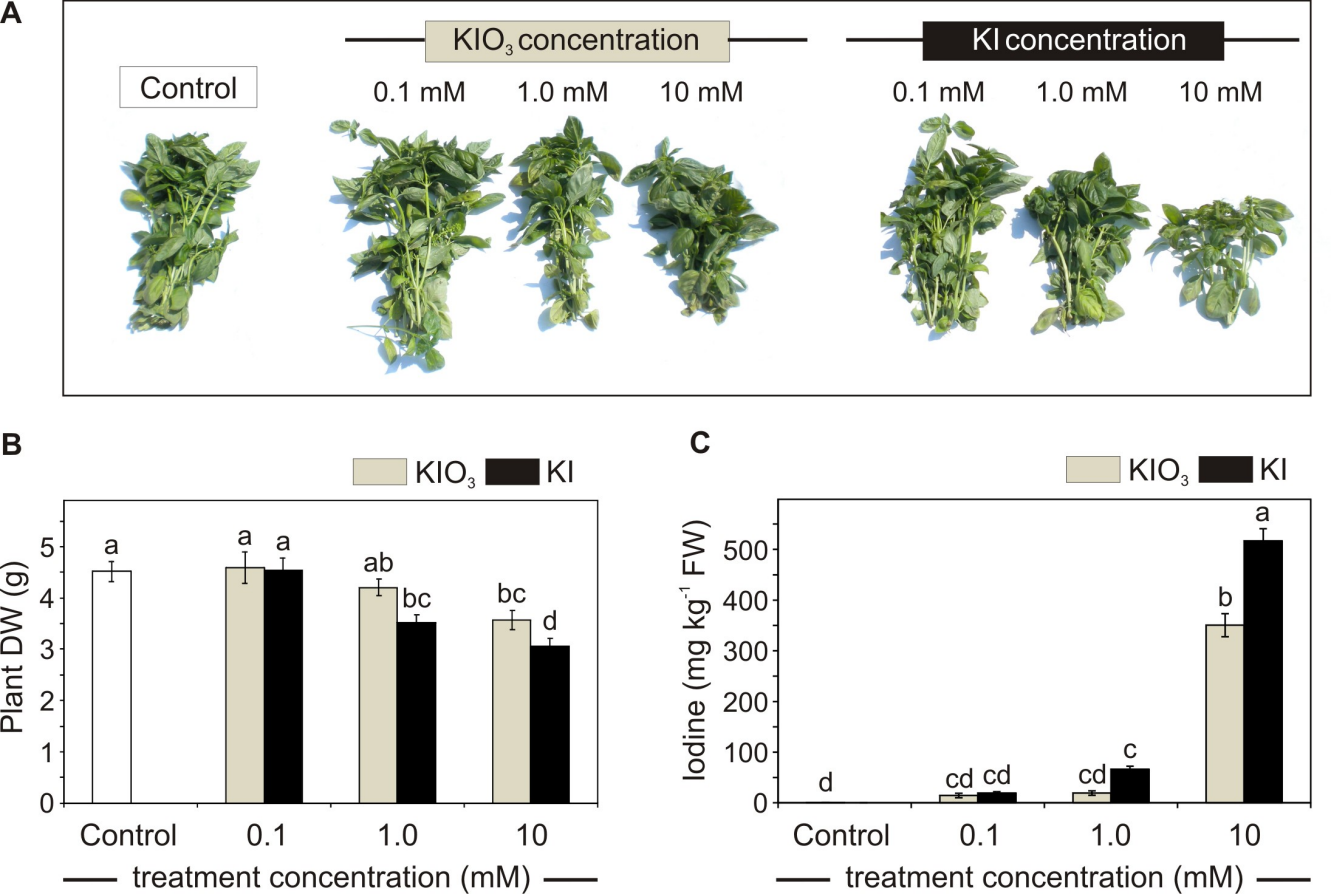
Effect of iodine on plant growth in open field experiment. Basil phenotype of control and KI or KIO_3_ (0, 0.1, 1.0 and 10 mM) treated plants at harvest (A); effects of increasing iodate od iodide concentrations on basil dry weight (DW) (B) and leaf iodine content (C). Error bars are presented on graphs. One-way ANOVA was performed. Different letters indicate significant differences between treatments (Tukey’s test, *P*≤0.05).

Iodine affected the plant growth, with a clear reduction of the plant size (Fig 1A) and biomass accumulation (Fig 1B) observed in a concentration-dependent manner, starting from 1.0 mM KI treatment and at 10 mM KIO_3_.

Moreover, the application of increasing concentrations of iodine induced some phytotoxicity symptoms, such as leaf abscission and the slight occurrence of brown necrotic areas in the upper leaves, especially in KI treated plants (S1 Fig). Nevertheless, no clear symptoms of chlorosis were present on the leaves, as demonstrated by the chlorophyll content, slightly affected only in 10 mM KI treated plants (Table 1).

**Table 1:** Effect of KIO_3_ and KI treatments (0, 0.1, 1.0 and 10 mM) on leaf chlorophyll content, chlorophyll a/b ratio, and carotenoids.

Treatment	Chlorophyll a+b		Chlorophyll a/b		Carotenoids	
	(mg/100g FW)		ratio		(mg/100g FW)	
Control	125.9 ± 11.3	AB	1.67 ± 0.09	n.s.	37.0 ± 0.45	n.s.
KIO_3_ 0.1 mM	129.3 ± 0.41	A	1.83 ± 0.19	n.s.	35.9 ± 1.2	n.s.
KIO_3_ 1.0 mM	118.7 ± 5.34	AB	1.73 ± 0.19	n.s.	32.6 ± 0.7	n.s.
KIO_3_ 10 mM	128.9 ± 7.56	A	1.43 ± 0.18	n.s.	31.9 ± 1.6	n.s.
KI 0.1 mM	126.3 ± 6.11	A	1.90 ± 0.06	n.s.	35.2 ± 2.4	n.s.
KI 1.0 mM	123.1 ± 7.28	AB	2.17 ± 0.09	n.s.	34.4 ± 1.4	n.s.
KI 10 mM	93.8 ± 2.38	B	2.17 ± 0.34	n.s.	30.4 ± 1.9	n.s.

Data were subjected to one-way ANOVA. Means within a column followed by different letters are significantly different (P≤0.05 according to Tukey’s test). Four different biological replicates were analyzed; standard error is reported in table.

The basil chlorophyll content (averaging 120.86 mg 100 g^-1^ FW) was similar to levels reported in literature [34]. Reduction of this parameter is generally associated to a physiological stressful condition, such as high salinity levels [35,36] or low nitrogen nutrition [37]. In addition, no statistically significant differences were found in the chlorophyll a/b ratio and carotenoids content (Table 1).

The effect of iodine on plant growth depends on the species undergoing biofortification and on the iodine form, concentration and cultivation system used [7,10]. Although high concentrations of iodine can be phytotoxic, contributions of up to 10 mg kg^-1^ in soil or a concentration lower than 80 μM KI/KIO_3_ can lead to positive effects on plant growth [7,17]. In the present study, the plant DW was reduced by approx. 20% and 30% compared to the control, respectively in IO_3_^—^ and I^—^treated plants, when applied at the maximum concentration (10 mM) (Fig 1B). The detrimental effect of iodine was more pronounced in KI treated plants due to the higher toxicity of iodides (I^-^) compared to iodate (IO_3_^-^) ions [7,10]. The higher phytotoxicity of I^-^ is probably due to fact that IO_3_^-^ is efficiently reduced to I^-^ before root absorption [13], and it may also represent a possible substrate for certain enzymes, such as nitrate reductases [38]. No negative effects on growth were observed in 0.1 mM KIO_3_/KI treated plants or in the presence of treatments with 1 mM KIO_3_.

The levels of accumulation of the element in basil leaves increased using both the forms of iodine in a dose-response manner (Fig 1C). Although soil treatment with iodine is preferentially done using KIO_3_ because the oxidized iodine form is likely to have a slower turnover compared to I^-^ in the soil, basil treatment with KI was the best way to increase iodine concentration in basil leaf tissues using lower iodine doses (Fig 1C). This fact is consistent with studies on plants grown in hydroponic systems, where root absorption seemed to prefer iodide compared to iodate. The analysis of leaf iodine content showed that a plausible treatment for biofortification purposes would be the use of KI between 0.1 mM and 1 mM, as the iodine content of leaves could fit into the desired range of concentrations established for human beings [39]. For instance, consuming 1g of 1mM KI biofortified basil (corresponding approx. to one leaf FW) would lead to an iodine intake of 67 μg, which represent the 44.6% of the daily recommended dietary allowance for an adult. The use of biofortified leaves characterized by higher iodine concentrations may be theoretically harmful for the consumer. Nevertheless, additional research on iodine bioavailability should be advisable.

To verify the possible effects of iodine treatments on the main qualitative basil traits, different biochemical determinations were performed on leaves, the organ of commercial interest.

Total phenolics, leaf antioxidant power and the concentration of selected CADs were measured to check which iodine form and concentration could be able to improve plant nutraceutical properties.

**Fig 2:**
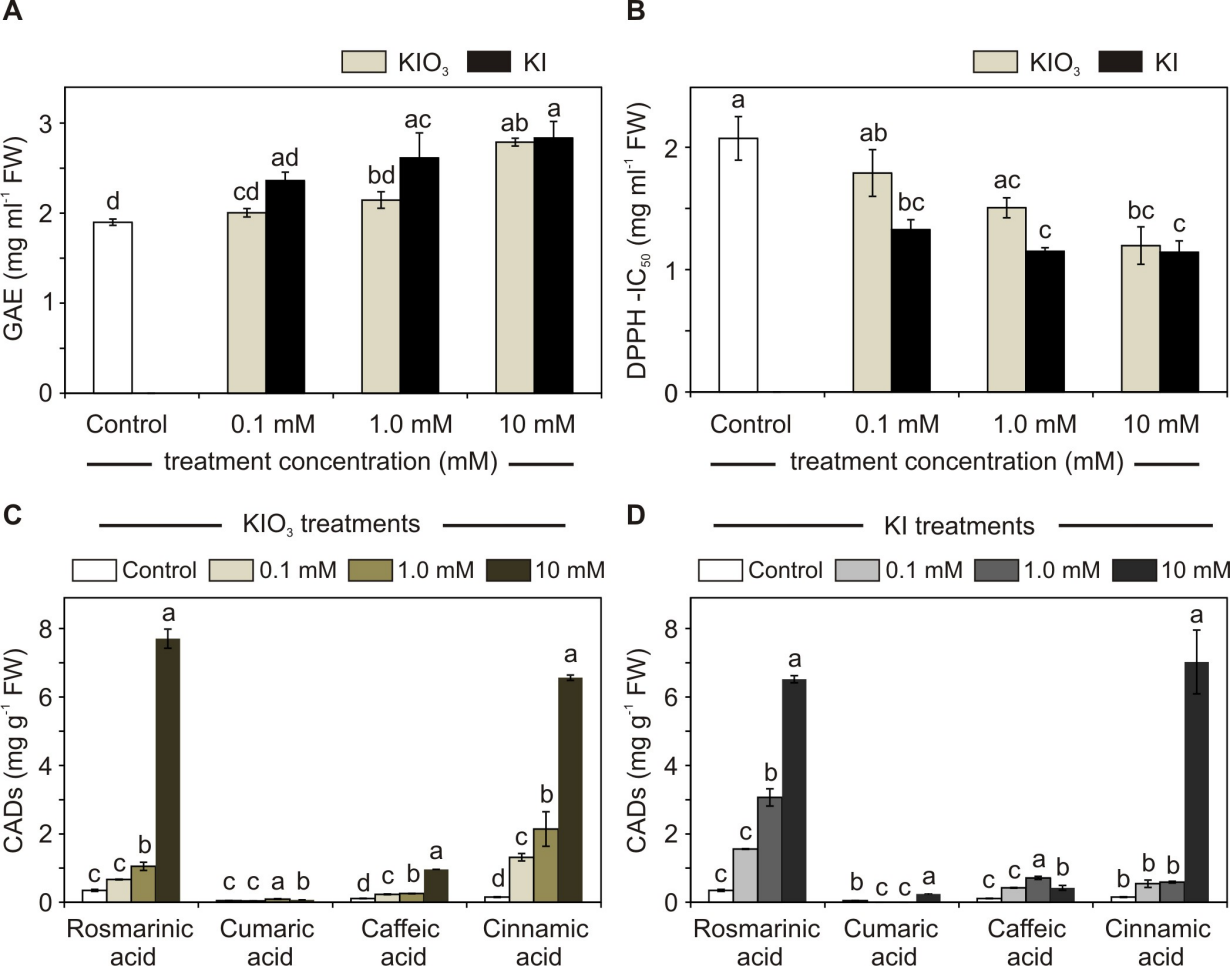
Phenolics and antioxidant power of basil plants grown in open field. Total phenolics (A) and antioxidant power (B) of control and KI or KIO_3_ (0, 0.1, 1.0 and 10 mM) treated plants. CADs of interest quantified in KIO_3_ (C) and KI (D) treated plants. Determinations were performed on basil leaves. Error bars are presented on graphs. One-way ANOVA was performed. Different letters indicate significant differences between treatments (Tukey’s test, *P*≤0.05).

Total phenols (Fig 2A) as well as the antioxidant power (Fig 2B) were significantly increased by treatments in a dose-response manner.

Compared to the control, the highest KI/KIO_3_ concentration used (10 mM) increased the GAE content approximately by 50% and almost doubled the leaf antioxidant power (IC50 value, which indicate the sample concentration required to scavenge 50% of the initial DPPH concentration, was reduced approx. by half). Iodide seemed to stimulate phenolic compounds accumulation and the antioxidant response more efficiently than iodate, as already demonstrated for tomato and lettuce plants [14,40]. Among phenolics, the accumulation of selected CADs, such as caffeic, ferulic, *p*-cumaric, chlorogenic, cinnamic and rosmarinic acids, the last one representing the most abundant and biologically active compound in basil [9,34,41], was deeply characterized. A positive correlation between iodine treatments and some CADs was found (Fig 2C and Fig 2D). The application of both 0.1 mM KI or KIO_3_ did not produce any significant differences in CADs production (Fig 2C and Fig 2D). On the contrary, rosmarinic and cinnamic acids production was strongly induced by KI or KIO_3_, especially at the highest concentration used (10 mM), as their accumulation was respectively increased approx. by 20 and 45-fold, in comparison with the controls. Rosmarinic and cinnamic acids are considered active principles having a broad spectrum of biological activities, including antiviral, antimicrobial and antifungal properties [42,43] and the large accumulation of these compounds increases the nutraceutical value of edible plants. These findings are in agreement with those reported in a previous work on hydroponically grown lettuce [17], where the application of low concentrations of IO_3_^-^, especially at rates of 20 and 40 μM, induced a significant increase in hydroxycinnamic acids, which seemed to contribute to the plant protection against salt stress.

Moreover, iodine treatments slightly induced the accumulation of cumaric and caffeic acids (Fig 2C and Fig 2D), whereas chlorogenic and ferulic acids remained below the detection limit (1 ng).

Since basil plants have a high value on the food and pharmaceutical market also due to their essential oil profiles [21], the extraction and the phytochemical characterization of the EO compositions under all the tested iodine treatments were performed.

The composition of the EO extracted from the open field basil plants are reported in Table 2.

**Table 2:** Complete compositions of the essential oils extracted from basil plants cultivated in open field after KI or KIO_3_ (0, 0.1, 1.0 and 10 mM) treatments.

Compounds	l.r.i.^1^	Relative abundance (%) ± standard deviation						
		Control	KI 0.1 mM	KI 1 mM	KI 10 mM	KIO_3_ 0.1 mM	KIO_3_ 1 mM	KIO_3_ 10 mM
α-pinene	941	0.5±0.01	0.1±0.00	0.2±0.00	-^2^	-	-	0.6±0.01
sabinene	976	0.4±0.00	0.1±0.00	0.3±0.23	0.2±0.00	0.2±0.00	-	0.5±0.00
β-pinene	982	1.1±0.00	0.4±0.00	0.2±0.35	-	-	0.2±0.00	1.3±0.00
myrcene	993	0.7±0.01	0.3±0.01	0.3±0.00	-	0.1±0.00	-	0.9±0.01
α-terpinene	1018	-	0.2±0.00	-	-	-	-	-
limonene	1032	0.3±0.00	-	-	-	-	-	-
**1,8-cineole**^**3**^	1034	13.0±0.02^B^	9.5±0.04^D^	12.0±0.04^C^	7.3±0.10^F^	8.0±0.04^E^	3.7±0.07^G^	14.6±0.04^A^
*cis*-sabinene hydrate	1070	0.2±0.00	0.2±0.00	0.3±0.00	0.3±0.01	0.3±0.01	0.2±0.00	0.2±0.00
*trans*-linalool oxide (furanoid)	1076	0.3±0.00	0.3±0.00	0.2±0.00	0.2±0.00	0.2±0.00	0.1±0.09	0.2±0.01
*cis*-linalool oxide (furanoid)	1077	0.2±0.00	0.2±0.00	0.2±0.00	0.2±0.01	0.2±0.00	-	0.2±0.00
**linalool**	1101	48.8±0.19^D^	52.9±0.20^C^	53.6±0.08^B,C^	54.5±0.88^B^	55.5±0.23^A^	36.8±0.65^F^	41.8±0.12^E^
3-octanol acetate	1125	-	-	-	-	-	-	0.1±0.01
camphor	1143	0.3±0.00	0.5±0.00	0.4±0.00	0.6±0.01	0.4±0.01	0.3±0.01	0.4±0.00
*iso*borneol	1156	0.1±0.14	0.6±0.00	0.8±0.00	0.4±0.01	0.6±0.00	0.5±0.01	-
δ-terpineol	1167	0.3±0.01	-	-	0.4±0.02	-	-	0.3±0.36
4-terpineol	1178	0.3±0.00	0.2±0.00	0.2±0.00	0.2±0.00	0.2±0.01	-	0.2±0.01
α-terpineol	1189	1.2±0.00	1.3±0.01	1.5±0.01	1.6±0.03	1.4±0.01	1.2±0.01	1.4±0.01
octyl acetate	1214	0.2±0.00	0.2±0.00	0.1±0.01	0.1±0.01	0.1±0.00	0.1±0.08	0.2±0.00
carvone	1244	-	-	-	-	-	0.1±0.00	-
*iso*bornyl acetate	1285	1.4±0.00	1.3±0.01	1.3±0.00	1.3±0.02	1.5±0.01	1.2±0.01	1.5±0.02
thymol	1292	-	-	-	-	-	0.1±0.02	-
carvacrol	1298	0.4±0.00	0.2±0.00	-	0.1±0.00	0.3±0.00	0.6±0.01	-
δ-elemene	1340	-	-	0.1±0.08	-	-	-	-
*exo*-2-hydroxycineole acetate	1344	0.1±0.08	0.1±0.00	0.1±0.08	0.1±0.12	0.1±0.00	0.1±0.09	0.1±0.09
**eugenol**	1358	1.0±0.02^E^	1.6±0.06^D^	1.9±0.04^A^	1.6±0.05^D^	1.9±0.02^A,B^	1.8±0.03^B,C^	1.8±0.01^C^
α-copaene	1376	-	0.1±0.12	-	0.1±0.11	-	-	-
β-bourbonene	1384	-	0.2±0.04	-	0.1±0.04	-	-	-
(*E*)-β-damascenone	1384	0.1±0.00	-	-	-	0.1±0.00	0.2±0.00	0.2±0.01
β-elemene	1392	0.3±0.01	0.4±0.02	0.4±0.01	0.5±0.01	0.4±0.05	0.5±0.06	0.4±0.01
methyl eugenol	1403	0.6±0.00	0.7±0.01	1.2±0.05	0.9±0.07	0.8±0.07	1.1±0.02	0.8±0.08
*cis*-α-bergamotene	1416	0.1±0.00	0.1±0.09	0.1±0.08	0.1±0.11	-	0.2±0.00	-
β-caryophyllene	1420	0.3±0.02	0.2±0.09	0.1±0.02	0.3±0.01	0.2±0.04	0.3±0.01	0.2±0.03
***trans*-α-bergamotene**	1438	7.3±0.25^D^	8.0±0.22^C^	6.5±0.01^E^	8.7±0.35^B^	7.0±0.21^D^	9.7±0.06^A^	6.3±0.04^E^
(*Z*)-β-farnesene	1444	-	0.1±0.00	0.1±0.07	0.1±0.00	-	0.2±0.00	-
aromadendrene	1445	-	-	-	-	-	-	0.1±0.19
*cis*-muurola-3,5-diene	1447	-	-	-	-	-	-	0.1±0.20
α-humulene	1456	0.5±0.02	0.5±0.02	0.5±0.00	0.5±0.02	0.5±0.01	0.4±0.49	0.6±0.16
(*E*)-β-farnesene	1460	0.4±0.01	0.3±0.01	0.3±0.00	0.2±0.18	0.3±0.01	0.5±0.01	0.5±0.13
*cis*-muurola-4(14),5-diene	1462	0.4±0.01	0.4±0.01	0.4±0.00	0.5±0.01	0.4±0.00	0.6±0.01	0.5±0.09
α-acoradiene	1463	-	-	-	-	-	-	0.2±0.11
β-acoradiene	1465	-	-	-	-	-	-	0.3±0.13
γ-muurolene	1477	-	-	-	-	0.6±0.01	1.0±0.20	-
germacrene D	1478	0.8±0.01	0.8±0.01	0.7±0.01	0.8±0.01	-	1.0±0.13	1.0±0.08
β-selinene	1485	0.6±0.03	0.7±0.03	-	-	-	-	0.9±0.13
valencene	1492	-	-	-	0.1±0.08	-	0.4±0.28	-
α-selinene	1494	0.1±0.07	-	-	-	-	-	0.3±0.05
bicyclogermacrene	1496	0.3±0.04	0.3±0.04	0.3±0.00	0.3±0.02	0.4±0.02	0.4±0.23	0.7±0.11
**α-bulnesene**	1505	-^E^	0.8±0.04^D^	1.5±0.02^A^	0.9±0.04^C^	-^E^	-^E^	1.0±0.08^B^
β-bisabolene	1509	0.8±0.04	0.1±0.08	-	0.1±0.08	0.9±0.04	2.0±0.20	-
***trans*-γ-cadinene**	1513	2.5±0.00^C^	2.4±0.00^D^	2.0±0.01^G^	2.9±0.05^B^	2.3±0.00^E^	3.4±0.06^A^	2.2±0.00^F^
cubebol	1516	-	-	-	0.2±0.01	-	-	-
*trans*-calamenene	1524	-	0.2±0.00	0.2±0.00	0.3±0.01	0.3±0.08	0.4±0.02	0.2±0.01
δ-cadinene	1524	0.2±0.01	-	-	-	-	-	-
β-sesquiphellandrene	1525	0.4±0.01	0.2±0.29	0.3±0.00	0.4±0.00	0.3±0.08	0.5±0.01	0.4±0.00
eugenol acetate	1528	-	-	-	-	-	-	0.1±0.16
*cis*-sesquisabinene hydrate	1545	0.2±0.00	0.2±0.01	0.1±0.00	0.1±0.06	0.1±0.01	0.2±0.01	-
(*E*)-nerolidol	1565	0.2±0.00	0.1±0.01	0.1±0.00	0.1±0.00	0.1±0.00	0.3±0.01	0.2±0.01
**spathulenol**	1576	1.2±0.04^B^	1.2±0.13^B^	1.0±0.08^B^	1.0±0.12^B^	1.2±0.10^B^	2.3±0.20^A^	1.2±0.08^B^
caryophyllene oxide	1581	0.1±0.09	0.1±0.08	0.1±0.08	0.1±0.08	-	0.1±0.21	0.1±0.11
viridiflorol	1590	-	-	0.1±0.01	-	0.3±0.01	0.2±0.04	0.8±0.06
humulene epoxide II	1608	0.2±0.01	0.1±0.04	0.1±0.04	0.1±0.03	0.1±0.04	0.3±0.13	0.2±0.00
**1,10-*di*-*epi*-cubenol**	1614	1.2±0.01^B,C^	1.1±0.01^C,D^	1.0±0.03^D^	1.2±0.01^B,C^	1.2±0.01^B,C^	2.1±0.13^A^	1.3±0.00^B^
γ-eudesmol	1630	-	-	-	-	-	-	0.1±0.01
***epi*-α-cadinol**	1640	9.0±0.16^B,C^	8.6±0.39^C^	7.1±0.11^D^	8.5±0.16^C^	8.9±0.30^C^	18.3±0.28^A^	9.5±0.33^B^
β-eudesmol	1650	0.2±0.03	0.2±0.11	0.2±0.10	0.2±0.08	0.2±0.11	0.7±0.29	0.3±0.10
α-cadinol	1654	0.6±0.09	0.6±0.10	0.5±0.09	0.5±0.08	0.6±0.11	1.4±0.30	0.6±0.07
bulnesol	1666	0.2±0.04	0.2±0.04	-	-	0.2±0.03	0.4±0.09	-
β-bisabolol	1672	-	-	0.2±0.04	-	-	-	0.7±0.08
α-bisabolol	1683	0.4±0.01	0.3±0.03	0.3±0.08	0.2±0.01	0.3±0.04	0.9±0.09	0.4±0.05
(*E*)-nerolidol acetate	1713	0.2±0.00	-	-	-	0.1±0.01	0.5±0.03	0.2±0.01
(*Z*)-β-santalol	1718	-	0.1±0.10	-	-	-	-	-
**Monoterpene hydrocarbons**		3.0±0.00^B^	1.1±0.01^C^	1.0±0.11^D^	0.2±0.00^F^	0.4±0.00^E^	0.2±0.00^F^	3.3±0.01^A^
**Oxygenated monoterpenes**		66.5±0.03^C^	67.2±0.25^C^	70.7±0.21^A^	67.0±0.93^C^	68.7±0.30^B^	44.9±0.93^E^	60.8±0.35^D^
**Sesquiterpene hydrocarbons**		15.0±0.06^C^	15.8±0.68^B,C^	13.3±0.06^D^	16.8±0.30^B^	13.6±0.21^D^	21.3±0.08^A^	16.0±1.44^B,C^
**Oxygenated sesquiterpenes**		13.3±0.02^C^	12.7±0.04^C,D^	10.8±0.21^E^	12.3±0.37^D^	13.3±0.04^C^	28.8±0.86^A^	15.6±0.08^B^
**Apocarotenoids**		0.1±0.00^A^	-^A^	-^A^	-^A^	0.1±0.00^A^	0.2±0.00^A^	0.2±0.01^A^
**Phenylpropanoids**		1.6±0.02^E^	2.3±0.04^D^	3.1±0.08^A^	2.6±0.12^C^	2.7±0.05^C^	2.9±0.05^B^	2.6±0.08^C^
**Other non-terpene derivatives**		0.2±0.00^B^	0.2±0.00^B^	0.1±0.01^C^	0.1±0.01^B,C^	0.1±0.00^C^	0.1±0.08^C^	0.3±0.01^A^
Total identified (%):		99.8±0.01	99.3±0.35	99.2±0.38	99.0±0.40	98.9±0.00	97.1±1.81	98.7±1.79

^1^ Linear retention indices on a HP-5MS column

^2^ Not detected

^3^ For compounds reported in bold and chemical classes, along the same row, different superscript uppercase letters (A,B,C,D,E,F) indicate significant differences (Tukey’s HSD, P < 0.05) among the samples.

**Fig 3:**
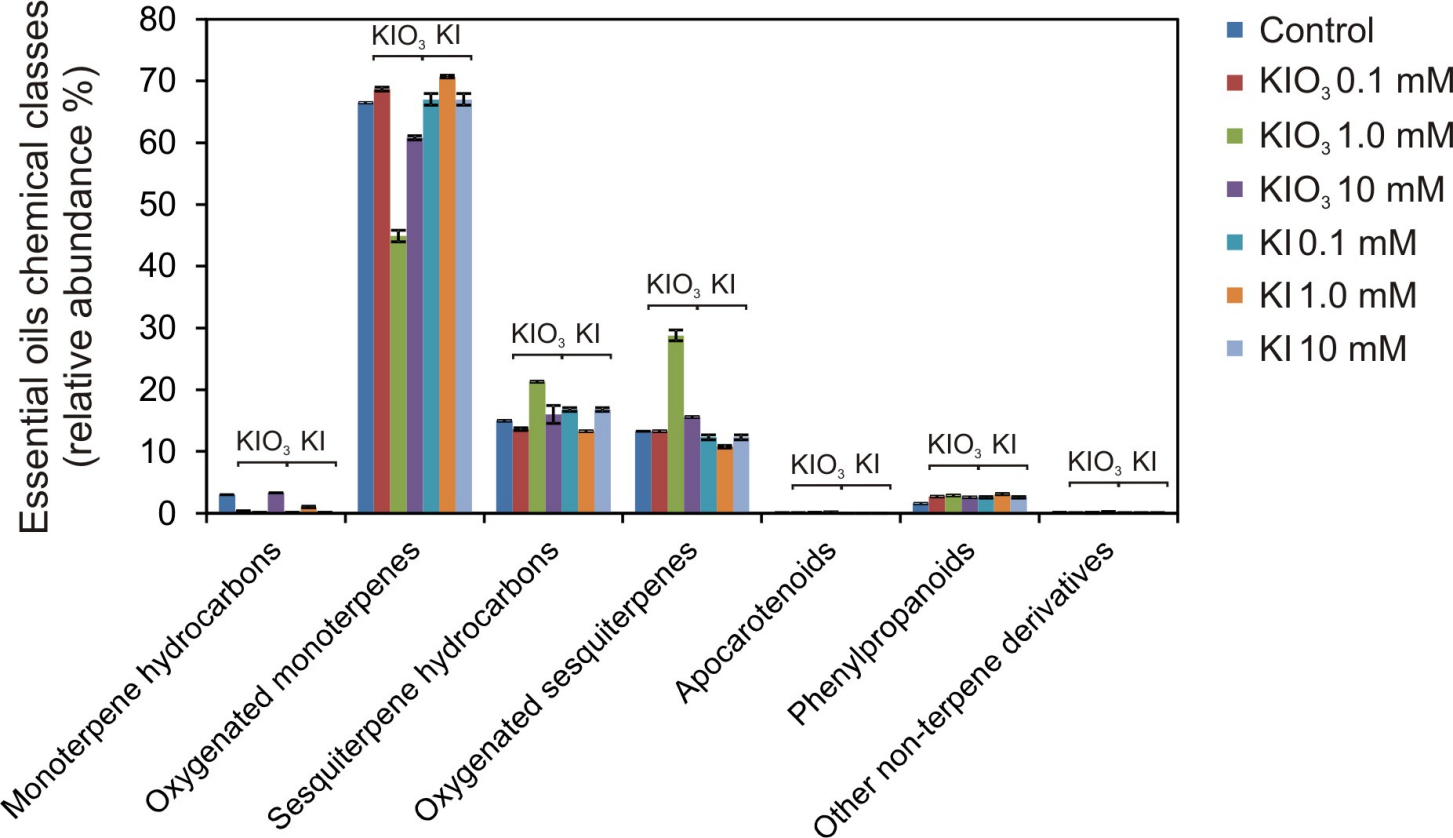
Essential oil composition of basil plants grown in open field. The behavior of the chemical classes of the volatile organic compounds was detected in the leaf essential oils extracted from basil samples treated with KI or KIO_3_ (0, 0.1, 1.0 and 10 mM).

The diagram in Fig 3 shows the behavior of the chemical classes of the detected compounds.

The oxygenated monoterpenes represented the most abundant chemical class, as they always accounted for more than 44% of the total EO composition, reaching more than 70% in the KI 1 mM treatment (Fig 3). The highest relative content of these compounds was detected in the KI 1 mM treatment, where they reached up to 70.7%. All the samples were characterized by a linalool chemotype, being this compound the most abundant oxygenated monoterpene, and the KIO_3_ 0.1 mM treatment induced its highest relative abundance (55.5%) (Table 2). The KIO_3_ 1 mM treated sample exhibited the lowest oxygenated monoterpenes relative content (44.9%), as well as the lowest linalool relative abundance (36.8%). Linalool is one of the main chemotypes reported in the literature for basil, together with the phenylpropanoid (chavicol and/or methyl chavicol) ones [44–47], which, however, in this experiment were not represented. 1,8-Cineole followed as the second most abundant oxygenated monoterpene, which reached relative abundances over 10% in the control, KI 1 mM and KIO_3_ 10 mM samples (Table 2).

Sesquiterpenes followed as the most detected class, with similar relative abundances for the hydrocarbon and oxygenated forms (Fig 3). Among the sesquiterpene hydrocarbons, *trans*-α-bergamotene and *trans*-γ-cadinene were the most represented compounds: both showed their highest relative contribution in the KIO_3_ 1 mM sample (9.7% and 3.4%, respectively) (Table 2). Among the oxygenated sesquiterpenes, the highest relative contribution was exhibited by *epi*-α-cadinol, followed by spathulenol and 1,10-*di*-*epi*-cubenol (18.3%, 2.3% and 2.1%, respectively) (Table 2). As for the sesquiterpene hydrocarbons, the KIO_3_ 1 mM sample exhibited the highest relative abundance of all these compounds (Fig 3).

The monoterpene hydrocarbons represented the fourth class of EOs in the control, even if their relative contribution decreased in almost all the treatments. Finally, followed the phenylpropanoids, whose level was quite low in the control (1.6%), but increased in all the treatments, even if remaining lower than 3.5% (Fig 3). Eugenol was the most represented phenylpropanoid, followed by its methyl ether (Table 2).

With the exception of the 1 mM KIO_3_ treatment, which slightly differed from the other treatments, the distribution of the main EOs in the different chemical classes was quite similar between the control and the iodine treated plants (Fig 3). This suggested that iodine did not represent a major factor able to modify as a whole the composition of the EOs in open field basil plants and that other factors, probably linked to the environmental conditions, resulted predominant. This result is particularly interesting by an agronomic point of view, as the overall typical plant aroma was not negatively influenced by the biofortification protocol carried out in the open field and the formation of off-flavors or toxic compounds was excluded, making this technique safe and easy to be applied in commercial conditions.

### Growth chamber experiment

Although foliar applications of plant micronutrients on basil were performed in previous works using zinc (Zn) and iron (Fe) [48], underlining that micronutrients treatment can modify chemical classes and production yield of essential oils, no published study verified whether or not iodine could have an influence on the volatile oil production in basil. The previous preliminary results obtained in the open field trial suggested a limited effect of the iodine treatment. Therefore, an experiment testing EOs variation under iodine treatment was performed in a growth chamber, to avoid the influence of the open field environmental factors (e.g. climate, pests, insects, etc.). Moreover, a wider range of KI and KIO_3_ concentrations (0; 10 μM; 0.1 mM; 1 mM; 10 mM; 100 mM) was used, in order to better characterize the possible effects of the treatments on the EOs composition.

At harvest, before EOs analysis, the plant phenotype was characterized and the DW and leaf iodine content were determined, in order to validate the results obtained in open field, thus making comparable the data on EO composition of the two experiments.

**Fig 4:**
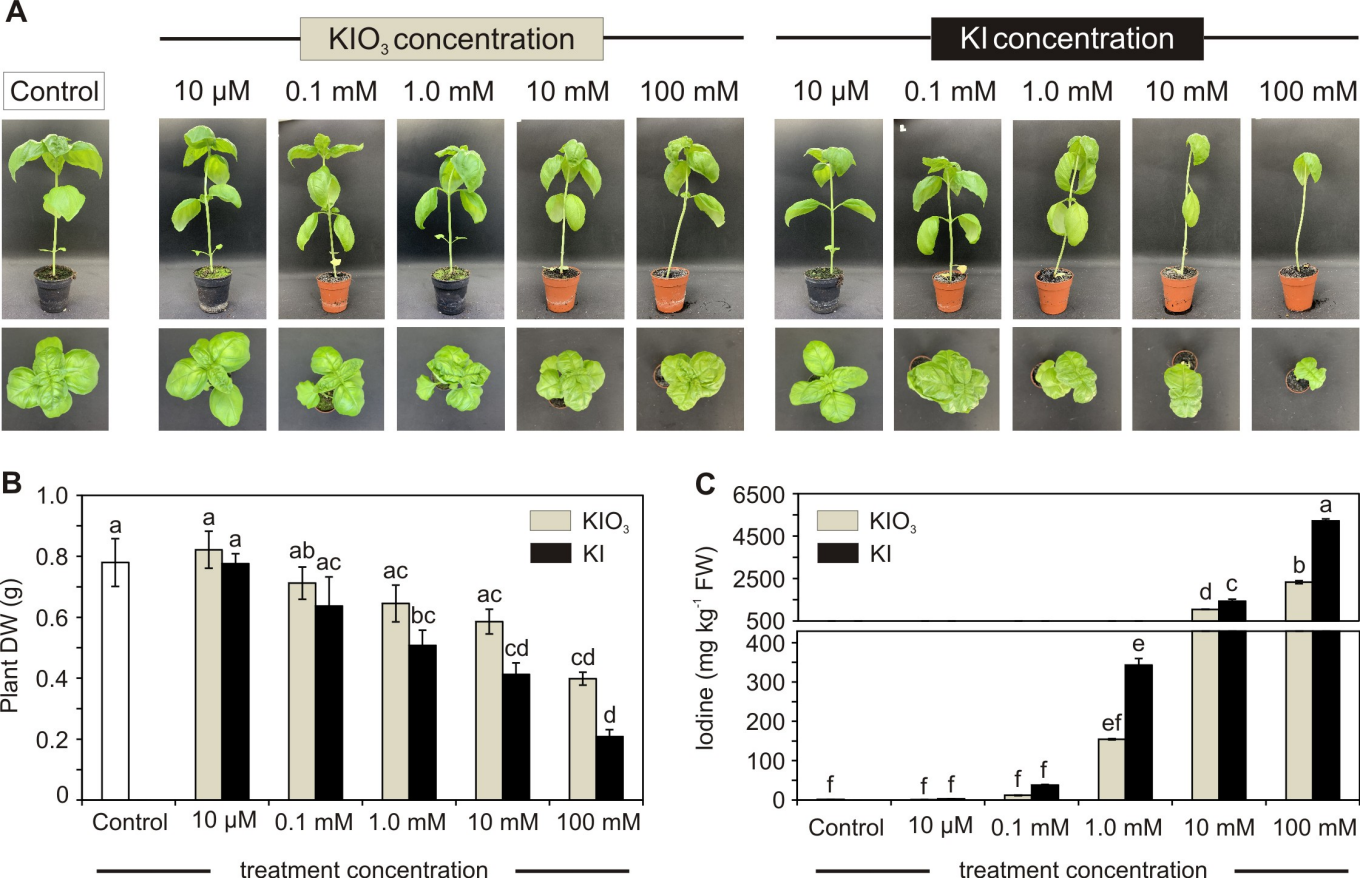
Effect of iodine on plant growth in growth chamber experiment. Basil phenotype of control and KI or KIO_3_ (0; 10 μM; 0.1 mM; 1 mM; 10 mM; 100 mM) treated plants at harvest (A); effects of increasing iodate and iodide concentrations on basil dry weight (DW) (B) and on leaf iodine content (C). Error bars are presented on graphs. One-way ANOVA was performed. Different letters indicate significant differences between treatments (Tukey’s test, *P*≤0.05).

The phytotoxic effect of iodine was evident on plants starting from 10 mM treatments and the most characterizing symptoms were leaf epinasty and abscission (Fig 4A), especially induced by KI.

Moreover, iodine phytotoxicity trend was confirmed by DW data where the detrimental effect of iodine and the higher toxicity of KI were validated. On the other hand, at 10 μM KIO_3_ a slight, even if no statistically significant, enhancement of plant DW was present (Fig 4B), supporting the hypothesis that low iodine concentration treatments would have beneficial effect on plants [7,17].

Data on iodine accumulation (Fig 4C) confirmed the trend observed in the open field experiment (Fig 1C): increasing treatment concentration enhanced iodine accumulation in leaves and the most efficient uptake of iodide compared to iodate was confirmed. At the same KI/KIO_3_ concentrations used, the iodine leaf content was mostly comparable between the two experiments, with the only exception of the 1 mM concentration, which, either in KI or in KIO_3_ treatment, induced a higher iodine accumulation in growth chamber-basil plants compared to the open field ones.

Treatments at the maximum dose (100 mM) scaled up iodine leaf accumulation, which ranged from 0.2 mg kg^-1^ in control plants to 2330 and 5215 mg kg^-1^ respectively in KIO_3_ and KI treated plants. These amounts exceeded by far the daily intakes recommended for iodine but demonstrated that basil can uptake and translocate the element in a very efficient way, even if it may be detrimental for plant growth and survival.

The complete composition of the EOs extracted from the growth chamber samples are reported in Table 3.

**Table 3:** Complete compositions of the essential oils extracted from basil plants cultivated in growth chamber after KI or KIO_3_ (0; 10 μM; 0.1 mM; 1 mM; 10 mM; 100 mM) treatments.

Compounds	l.r.i.^1^	Relative abundance (%) ± standard deviation										
		Control	KI 0.01 mM	KI 0.1 mM	KI 1 mM	KI 10 mM	KI 100 mM	KIO_3_ 0.01 mM	KIO_3_ 0.1 mM	KIO_3_ 1 mM	KIO_3_ 10 mM	KIO_3_ 100 mM
(*Z*)-3-hexen-1-ol	857	-^2^	0.1±0.13	0.1±0.03	0.1±0.09	-	-	-	0.1±0.00	0.1±0.01	0.2±0.01	-
α-pinene	941	-	-	-	-	-	-	-	-	-	-	0.1±0.01
sabinene	976	-	-	-	-	-	0.2±0.01	-	-	-	-	0.2±0.01
1-octen-3-ol	980	0.1±0.01	-	0.2±0.03	0.1±0.07	-	-	-	-	-	0.1±0.01	-
β-pinene	982	-	0.4±0.01	-	-	-	0.4±0.00	0.1±0.12	0.1±0.01	-	-	0.5±0.01
myrcene	993	-	0.2±0.01	0.1±0.07	-	-	0.4±0.01	0.1±0.07	0.1±0.07	-	-	0.4±0.01
limonene	1032	-	0.2±0.01	0.1±0.08	-	-	0.3±0.00	0.1±0.00	-	0.1±0.01	-	0.3±0.00
**1,8-cineole**^**3**^	1034	4.1±0.46^D,E^	6.7±0.12^A^	2.9±0.44^B^	1.8±0.18^F,G^	3.9±0.23^D,E^	8.2±0.50^C^	4.3±0.19^D^	2.5±0.01^F^	1.1±0.04^G^	2.9±0.13^E,F^	7.5±0.16^C^
(*E*)-β-ocimene	1052	0.4±0.04	0.9±0.02	0.7±0.15	0.4±0.02	0.2±0.05	0.6±0.01	0.9±0.03	0.7±0.08	0.3±0.00	-	1.6±0.03
*cis*-sabinene hydrate	1070	0.2±0.01	0.2±0.01	0.2±0.03	-	0.2±0.02	0.4±0.01	0.2±0.01	0.1±0.08	-	0.2±0.02	0.4±0.00
terpinolene	1088	-	0.2±0.00	0.1±0.03	-	-	0.2±0.01	0.2±0.01	-	-	-	0.2±0.00
**linalool**	1101	10.5±1.02^F^	23.0±0.63^B^	14.8±1.68^D^	8.4±0.76^G^	5.6±0.39^H^	17.5±0.76^C^	31.2±1.61^A^	12.4±0.30^E,F^	3.7±0.06^H^	12.7±0.18^E^	20.3±0.3
limona ketone	1130	-	-	-	-	0.6±0.06	0.2±0.01	-	-	-	0.2±0.02	0.1±0.00
camphor	1143	0.3±0.03	0.7±0.02	0.2±0.02	0.3±0.02	0.5±0.05	0.7±0.01	0.5±0.01	0.3±0.00	0.2±0.01	0.3±0.02	0.5±0.01
borneol	1165	0.8±0.09	0.6±0.02	0.7±0.09	0.3±0.02	0.4±0.19	0.3±0.07	0.7±0.01	0.3±0.07	-	0.5±0.04	0.8±0.01
δ-terpineol	1167	0.1±0.13	-	-	-	0.1±0.11	0.4±0.05	-	-	-	-	0.3±0.01
4-terpineol	1178	0.3±0.01	-	-	-	-	-	-	-	-	-	-
α-terpineol	1189	1.4±0.05	1.0±0.03	0.7±0.08	0.4±0.03	1.2±0.11	1.7±0.04	0.8±0.03	0.5±0.04	0.3±0.02	1.1±0.04	1.6±0.01
methyl salicylate	1192	-	-	-	-	-	0.2±0.01	-	-	-	-	-
*o*-cumenol	1199	-	-	-	-	0.2±0.02	-	-	-	-	-	-
octyl acetate	1214	-	-	-	-	-	0.1±0.01	-	-	-	-	-
bornyl acetate	1287	1.0±0.01	1.1±0.01	1.4±0.07	1.8±0.07	1.4±0.08	1.7±0.04	2.0±0.01	1.1±0.04	1.6±0.05	0.9±0.01	1.7±0.01
**eugenol**	1358	29.5±0.04^C^	16.0±0.01^E^	9.9±0.35^G^	11.1±0.13^F^	30.1±0.14^C^	27.1±0.07^D^	7.6±0.62^I^	8.8±0.59^H^	6.0±0.02^J^	37.2±0.14^A^	32.2±0.09^B^
α-copaene	1376	-	-	-	0.1±0.01	-	-	-	0.1±0.02	0.1±0.00	-	-
(*E*)-methyl cinnamate	1380	0.2±0.01	0.2±0.01	0.2±0.01	0.1±0.01	0.3±0.01	0.2±0.01	0.2±0.02	0.1±0.01	0.1±0.01	0.2±0.01	0.2±0.00
β-cubebene	1390	0.1±0.07	-	-	0.1±0.07	-	0.1±0.07	-	0.1±0.08	-	-	0.1±0.01
β-elemene	1392	0.1±0.04	0.5±0.05	0.4±0.11	0.6±0.11	0.1±0.11	0.1±0.13	0.5±0.13	0.6±0.05	0.6±0.18	0.3±0.03	0.2±0.02
**methyl eugenol**	1403	21.9±0.07^A^	20.2±0.35^D^	25.6±1.1^B^	15.7±0.54^F^	29.9±0.95^A^	9.7±0.01^G^	17.1±0.43^E,F^	15.9±0.92^F^	23.6±1.38^C^	18.2±0.33^E^	4.2±0.01^H^
*cis*-α-bergamotene	1416	-	-	-	0.1±0.00	-	-	-	0.1±0.01	0.1±0.00	-	-
β-caryophyllene	1420	0.2±0.00	0.5±0.02	0.4±0.01	0.4±0.00	0.2±0.01	0.2±0.01	0.4±0.02	0.4±0.03	0.8±0.02	0.2±0.00	0.2±0.00
***trans*-α-bergamotene**	1438	8.1±0.03^F^	8.2±0.02^F^	12.5±0.62^D^	16.9±0.19^C^	6.7±0.14^G,H^	6.4±0.01^H^	9.8±0.14^E^	18.3±0.75^B^	19.3±0.5^A^	7.2±0.27^G^	5.0±0.02^I^
α-guaiene	1439	0.2±0.02	0.3±0.01	0.4±0.03	0.8±0.03	0.2±0.00	0.4±0.00	0.3±0.00	0.7±0.02	0.6±0.01	0.3±0.02	0.5±0.00
(*Z*)-β-farnesene	1444	0.1±0.01	-	0.1±0.00	0.2±0.01	-	-	-	0.2±0.02	-	-	-
aromadendrene	1445	-	-	0.1±0.01	-	-	-	-	-	-	-	-
*cis*-muurola-3,5-diene	1447	0.1±0.02	-	-	0.2±0.00	-	0.2±0.01	-	-	0.1±0.00	-	0.2±0.00
**α-humulene**	1456	1.2±0.04^F^	1.6±0.04^E^	2.4±0.08^C^	2.7±0.01^B^	1.5±0.03^E^	1.2±0.04^F^	1.9±0.02^D^	2.7±0.06^B^	4.0±0.03^A^	1.5±0.01^E^	1.2±0.01^F^
**(*E*)-β-farnesene**	1460	3.8±0.04^F^	4.1±0.08^F^	6.1±0.30^D^	8.3±0.30^B^	4.7±0.01^E^	2.5±0.11^H^	4.6±0.25^E^	7.5±0.24^C^	12.0±0.3^A^	3.1±0.07^G^	2.8±0.03^G,H^
*cis*-muurola-4(14),5-diene	1462	0.2±0.01	0.2±0.01	0.3±0.06	0.5±0.07	0.2±0.01	0.3±0.05	0.3±0.01	0.5±0.04	0.4±0.01	0.2±0.06	0.3±0.04
**germacrene D**	1478	3.0±0.09^C^	2.9±0.11^C,D^	4.1±0.11^B^	5.8±0.11^A^	2.7±0.06^E,F^	2.8±0.07^D,E^	3.0±0.25^C,D^	5.6±0.08^A^	5.8±0.10^A^	2.5±0.03^F^	2.9±0.08^C,D,E^
β-chamigrene	1485	-	0.6±0.01	1.0±0.05	1.3±0.01	-	-	0.7±0.21	1.5±0.11	1.5±0.01	0.6±0.04	0.4±0.08
bicyclogermacrene	1496	1.1±0.04	1.2±0.07	1.8±0.08	2.5±0.07	1.0±0.01	1.2±0.04	1.4±0.01	2.1±0.08	2.2±0.01	0.9±0.01	1.4±0.01
**α-bulnesene**	1505	-^G^	1.3±0.04^E,F^	2.3±0.16^D^	3.7±0.04^A^	-^G^	-^G^	1.2±0.01^F^	2.5±0.04^C^	2.8±0.01^B^	1.4±0.02^E^	-^G^
germacrene A	1506	1.4±0.08	-	-	-	1.2±0.04	2.0±0.03	-	-	-	-	2.2±0.01
β-bisabolene	1509	0.2±0.04	0.1±0.07	0.1±0.00	0.1±0.09	0.3±0.04	0.1±0.01	0.1±0.02	0.2±0.01	0.4±0.04	0.2±0.00	0.1±0.01
***trans*-γ-cadinene**	1513	1.5±0.09^E^	1.5±0.03^E^	1.9±0.08^C^	3.1±0.04^A^	1.1±0.01^D^	1.8±0.05^G^	1.7±0.03^D^	3.0±0.08^A^	2.4±0.04^B^	1.4±0.01^F^	1.8±0.03^D^
7-*epi*-α-selinene	1517	-	-	-	0.1±0.01	-	-	0.1±0.01	0.2±0.01	0.2±0.00	-	-
β-sesquiphellandrene	1525	0.6±0.05	0.4±0.03	0.6±0.01	0.9±0.09	0.5±0.04	0.5±0.03	0.4±0.06	1.0±0.1	0.9±0.11	0.4±0.00	0.4±0.03
eugenol acetate	1528	-	-	-	-	-	0.1±0.01	-	-	-	-	-
selina-3,7(11)-diene	1542	-	-	-	0.2±0.01	-	0.2±0.01	-	-	-	-	0.1±0.08
*cis*-sesquisabinene hydrate	1545	-	-	-	0.1±0.08	-	0.1±0.07	-	0.1±0.08	0.1±0.08	-	-
(*E*)-nerolidol	1565	-	-	-	0.3±0.06	-	0.2±0.02	-	-	0.3±0.05	0.1±0.00	0.1±0.01
caryophyllene oxide	1581	-	0.2±0.01	-	-	-	-	0.2±0.04	-	0.2±0.04	-	-
viridiflorol	1590	-	-	-	-	0.2±0.03	0.3±0.02	-	-	-	-	0.2±0.01
1,10-*di*-*epi*-cubenol	1614	0.6±0.07	0.4±0.04	0.5±0.01	0.8±0.13	-	-	0.5±0.06	0.7±0.11	0.5±0.03	0.4±0.01	0.7±0.02
***epi*-α-cadinol**	1640	4.8±0.34^D^	3.9±0.21^E^	6.1±0.45^B^	8.0±0.57^A^	3.4±0.06^E^	6.2±0.08^B^	5.8±0.08^B,C^	7.4±0.09^A^	5.7±0.52^B,C^	3.9±0.01^E^	5.2±0.08^C,D^
β-eudesmol	1650	0.3±0.07	0.2±0.02	0.4±0.05	0.5±0.06	0.2±0.01	0.3±0.01	0.3±0.00	0.4±0.02	0.5±0.05	0.3±0.01	0.2±0.01
α-cadinol	1654	0.3±0.01	0.2±0.01	0.3±0.05	0.4±0.06	0.2±0.03	0.3±0.04	0.3±0.04	0.4±0.01	0.3±0.02	0.2±0.01	0.2±0.01
*neo*intermedeol	1660	0.1±0.03	-	-	-	0.2±0.02	-	-	-	-	-	-
14-hydroxy-9-*epi*-(*E*)-caryophyllene	1664	-	-	-	-	-	-	-	-	0.1±0.08	-	-
α-bisabolol	1683	-	-	-	0.1±0.16	0.3±0.06	0.3±0.01	-	0.1±0.12	0.1±0.20	0.3±0.01	0.1±0.02
methyl *p*-methoxycinnamate	1692	-	-	0.1±0.01	-	-	-	0.2±0.06	-	-	-	-
phytol	2114	0.3±0.36	-	-	0.1±0.02	-	0.2±0.02	-	0.1±0.08	0.2±0.06	-	-
*n*-pentacosane	2500	-	-	-	0.1±0.13	-	-	-	-	-	-	-
**Monoterpene hydrocarbons**		0.4±0.04^E^	1.8±0.00^B^	0.9±0.33^D^	0.4±0.02^E^	0.2±0.05^E,F^	2.0±0.03^B^	1.3±0.23^C^	0.9±0.17^D^	0.4±0.01^E^	-^F^	3.3±0.03^A^
**Oxygenated monoterpenes**		18.7±1.62^C,D^	33.3±0.66^B^	20.9±2.42^C^	13.0±1.09^E^	13.9±1.02^E^	31.0±1.14^B^	39.7±1.78^A^	17.1±0.52^D^	6.9±0.06^F^	18.9±0.47^C,D^	33.2±0.44^B^
**Sesquiterpene hydrocarbons**		22.0±0.56^E^	23.4±0.49^E^	34.5±1.43^C^	48.4±0.66^B^	20.2±0.18^F^	19.7±0.62^F^	26.3±0.91^D^	47.2±0.74^B^	54.2±0.33^A^	19.9±0.33^F^	19.7±0.22^F^
**Oxygenated sesquiterpenes**		6.0±0.52^C,D^	4.8±0.30^E^	7.3±0.54^B^	10.1±1.12^A^	4.5±0.21^E^	7.6±0.25^B^	7.1±0.22^B,C^	9.0±0.25^A^	7.7±1.02^B^	5.2±0.01^D,E^	6.8±0.16^B,C^
**Oxygenated diterpenes**		0.3±0.36^A^	-^B^	-^B^	0.1±0.02^A,B^	-^B^	0.2±0.02^A,B^	-^B^	0.1±0.08^A,B^	0.2±0.06^A,B^	-^B^	-^B^
**Phenylpropanoids**		51.7±0.11^C^	36.4±0.33^D,E^	35.8±0.74^E^	27.0±0.66^G^	60.2±1.08^A^	37.2±0.06^D^	25.0±0.27^H^	24.8±0.33^H^	29.7±1.41^F^	55.5±0.18^B^	36.7±0.11^D,E^
**Other non-terpene derivatives**		0.1±0.01^B,C^	0.1±0.13^B,C^	0.3±0.06^A,B^	0.2±0.29^A,B,C^	0.2±0.02^A,B,C^	0.4±0.02^A^	-^C^	0.1±0.00^B,C^	0.1±0.01^B,C^	0.3±0.02^A,B^	-^C^
Total identified (%):		99.2±0.11	99.8±0.06	99.7±0.10	99.2±0.26	99.2±0.04	98.1±0.29	99.5±0.16	99.2±0.04	99.2±0.09	99.8±0.01	99.7±0.01

^1^ Linear retention indices on a HP-5MS column

^2^ Not detected

^3^ For compounds reported in bold and chemical classes, along the same row, different superscript uppercase letters (A,B,C,D,E,F) indicate significant differences (Tukey’s HSD, P < 0.05) among the samples.

**Fig 5:**
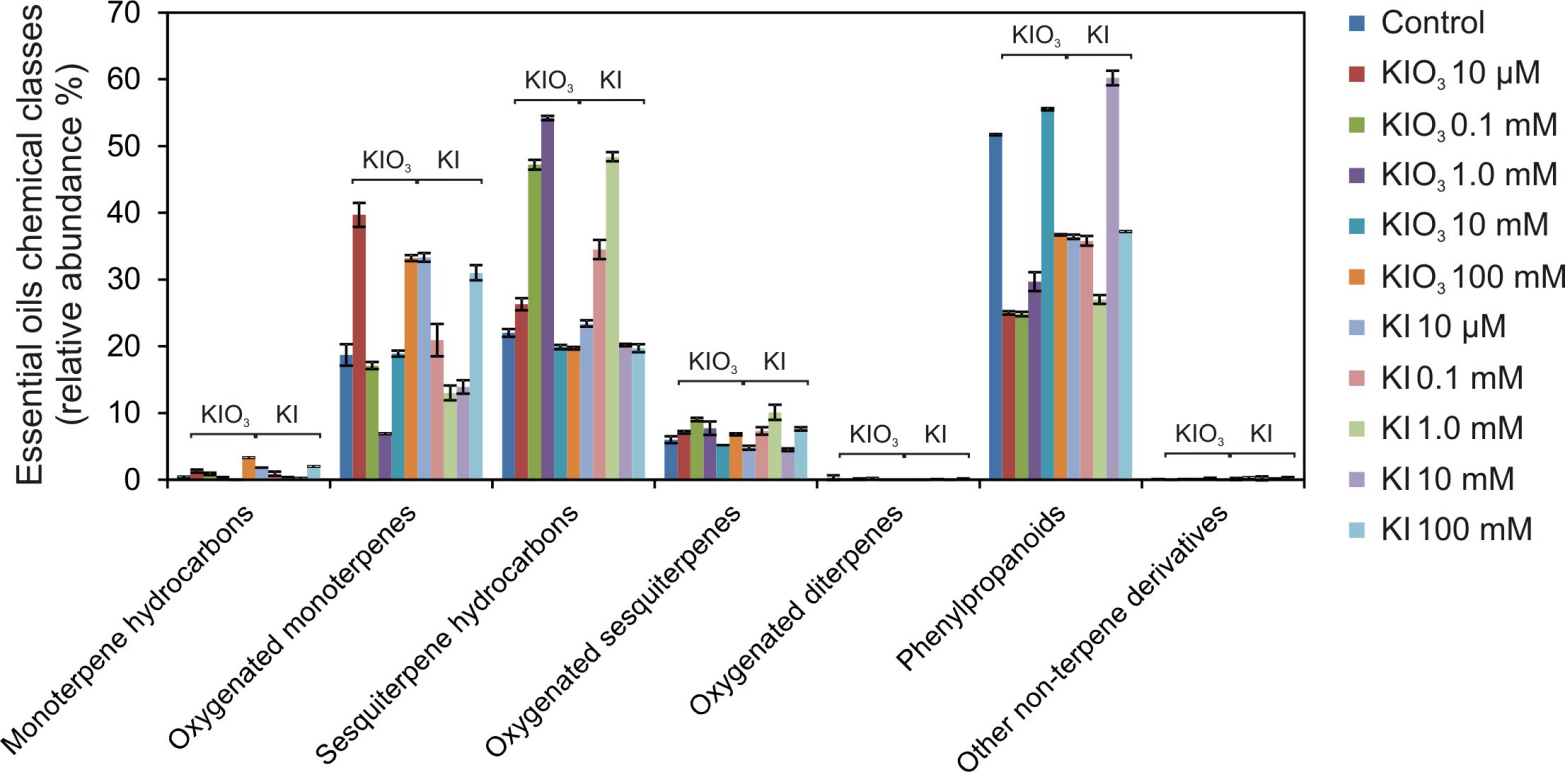
Essential oil composition of basil plants cultivated in growth chamber. The behavior of the chemical classes of the volatile organic compounds was detected in the compositions of leaf essential oils extracted from basil plants treated with KI or KIO_3_ (0; 10 μM; 0.1 mM; 1 mM; 10 mM; 100 mM).

The diagram in Fig 5 shows the behavior of the chemical classes of the detected compounds.

The chemical classes identified in the EOs extracted from the specimens of the growth chamber showed a larger variability among the different treatments (Fig 5), compared to the open field experiment (Fig 3). The effect of the controlled environment of the growth chamber compared to the open field situation is already evident in the control sample, which showed a chemotype switch from linalool (Table 2) to eugenol/methyl eugenol (Table 3). This change was significant, since eugenol and methyl eugenol alone accounted for more than 50% of all the volatile compounds produced from control basil plants in the growth chamber. We can speculate that this important change in the EOs production has to be linked to a completely different environment where plants had to grow. In the open field, indeed, plants need to face different kinds of possible biotic and abiotic attacks; therefore, the pattern of the EOs produced must be carefully calibrated towards such a competitive environment. An increment in linalool has been reported as a response to plant parasites and herbivores [49,50]. This evidence seems in accordance with the compositions evidenced in the present study: linalool was higher in the open field plants presumably as a protective metabolite against herbivores and parasites, which were not present in the growth chamber.

Due to the high concentration of eugenol and methyl eugenol, phenylpropanoids were detected as the most represented compounds (51.7%) in the control plants cultured in the growth chamber (Fig 5). The same occurred in all the KI-treated specimens (with the exception of the 1 mM concentration) and in samples treated with the two highest concentrations (10 and 100 mM) of KIO_3_ (Fig 5). Interestingly, treatments with both iodine forms at the 10 mM concentration induced the highest relative abundances of these compounds, closely followed by the control basil, with a statistically significant difference compared to all the other plants (Table 3). Moreover, these three samples all showed a eugenol/methyl eugenol chemotype (Table 3).

Further increasing the iodine concentration resulted in a reduction of the methyl eugenol production with the concomitant increase of the linalool fraction and a change of the chemotype: the EOs of the basil treated with the 100 mM concentration of both the iodine forms exhibited indeed a eugenol/linalool chemotype, with the former being slightly more abundant (Table 3). Conversely, the lowest iodine concentrations of both KI and KIO_3_ induced a reduction of the eugenol fraction with the advantage of the linalool one and thus a linalool/methyl eugenol chemotype (KI 0.01 mM) or linalool-only chemotype (KIO_3_ 0.01 mM) (Table 3). These findings seem in accordance with the hypothesis of linalool being a stress-induced compound in basil, as such a condition could be attributed to both the competitiveness of the open field environment and the phytotoxic levels of iodine reached when administered in the highest concentrations. Only plants treated with 0.1 mM of KI exhibited a methyl eugenol-only chemotype (Table 3). Sesquiterpene hydrocarbons were identified as the second class of volatile compounds in control plants (22%), but they were also found in concentrations higher than 45% of the total EO composition in the samples treated with KI 1 mM, and with KIO_3_ 0.1 and 1 mM (Fig 5). These three samples, indeed, exhibited a *trans*-α-bergamotene/methyl eugenol chemotype, with the former detected in statistically relevant higher concentrations (Table 3).

A relevant higher presence of oxygenated monoterpenes was observed in the EO of the KIO_3_ 0.01 mM-treated sample (Table 3): the latter was, indeed, as already stated, the only basil exhibiting a linalool-only chemotype among the growth chamber ones. Linalool was the most represented oxygenated monoterpene also in control plants and in all the other samples, followed by 1,8-cineole (Table 3).

Oxygenated sesquiterpenes and monoterpene hydrocarbons represented the following classes in control basil plants developed in the growth chamber, with small differences in the iodine-treated groups (Table 3 and Fig 5).

The significant changes in the distribution of the main EO classes and, within them, of the single compounds, which were observed in the growth chamber iodine-treated plants, both among them and in comparison with the control, is worth of further studies. Particularly significant was the “association” between some ranges of iodine concentrations and some EO chemotypes, which, if confirmed, could represent a system to be exploited to drive the basil production of specific EO constituents for particular industrial applications (e.g. perfumes, cosmetics, flavoring, etc).

### Multivariate statistical analysis on the essential oil compositions of all the samples

**Fig 6:**
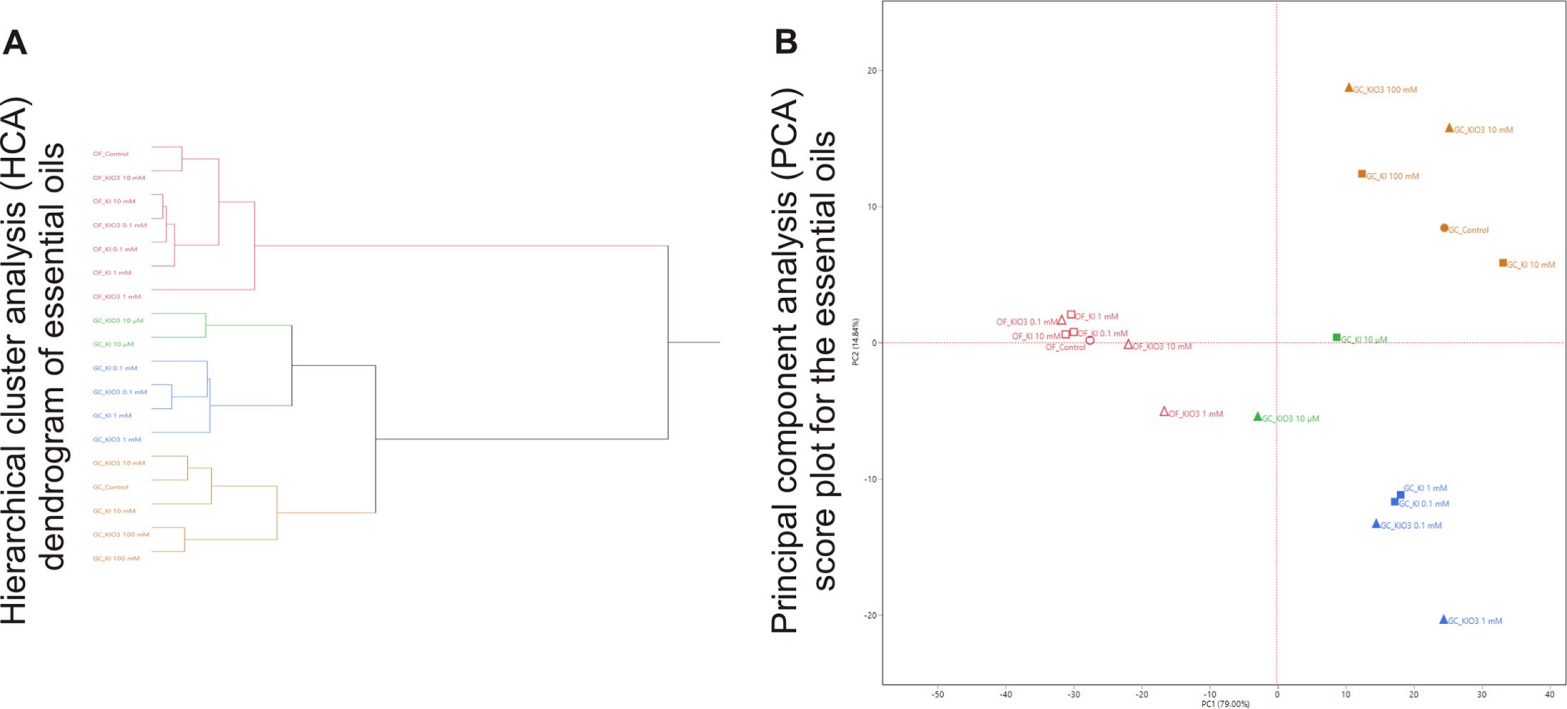
Multivariate statistical analysis on the essential oil compositions of all the samples. Hierarchical cluster analysis (HCA) dendrogram (A) and principal component analysis (PCA) score plot (B) for the complete compositions of leaf essential oils extracted from the open field and growth chamber samples.

The dendrogram of the hierarchical cluster analysis (HCA) performed on the complete compositions of the EOs extracted from the open field and the growth chamber samples combined is reported in Fig 6A.

In the HCA dendrogram, the first macro-clustering sharply divided the open field samples from the growth chamber plants (Fig 6A): this was not surprising, as all the open field basil EOs showed the linalool chemotype (Table 2), while the specimens of the growth chamber exhibited a large variability in their volatile oil compositions (Table 3). The macro-cluster aggregating the growth chamber basil EOs composition was further divided into three sub-clusters (Fig 6A). The green and the blue sub-clusters group the lowest (10 μM) and intermediate (0.1 mM and 1 mM) concentrations of both the iodine treatments; the orange one, instead, clusters the control together with the highest (10 and 100 mM) iodine concentrations.

This pattern was confirmed in the score plot of the PCA analysis (Fig 6B), in which all the open field samples were positioned in the left quadrants (PC1<0): the loadings plot evidenced the higher contribution of oxygenated monoterpenes and, in particular, linalool for the plotting of these samples. The growth chamber basil EOs, instead, were plotted in the right quadrants (PC1>0) in a more scattered fashion (Fig 6B), testifying the higher variability of their compositions. In any case, it was evident that the distribution of the three HCA sub-clusters aggregating the growth chamber samples was maintained. The bottom right quadrant (PC1>0, PC2<0) contained the samples treated with the intermediate concentrations (0.1 and 1 mM) of both the iodine forms (blue sub-group), as they exhibited a higher relative contents of sesquiterpenes, as shown in the loadings plot for the PC analysis (S2 Fig).

The basil EOs of the samples treated with the lowest concentration (10 μM) were plotted between the upper and the lower section of the upper right quadrant, in the closest position to the open field samples compared to all the other specimens of the growth chamber (Fig 6B). This was due to their higher content of oxygenated monoterpenes (S2 Fig) compared to all the other plants treated in this second experiment. Finally, the orange sub-group of the control and the EOs of the basil treated with the higher concentrations (10 and 100 mM) of iodine was plotted in the upper right quadrant (PC1 and PC2>0): as evidenced in the loadings plot, the high phenylpropanoid content of these samples is the reason for their positioning in this area (S2 Fig).

If we hold true that low iodine levels can be beneficial for plants due to some not yet characterized physiological effect of this element, its absence or insufficiency could represent a negative and stressful condition for the plant. This could in some way explain why control and iodine-treated plants with the highest and phytotoxic concentrations grouped together, distinguishing from the other two groups that included the low and the intermediate iodine concentrations.

## Conclusions

In our study, basil emerged as a good target for iodine biofortification, as the plant was able to efficiently uptake and translocate large amounts of the element, which were stored in the leaves in a dose-dependent manner. The higher assimilation rate of iodide comparing to iodate was confirmed in both the open field and the growth chamber trials, and, following the adopted biofortification protocol, the optimal concentrations to be used should range between 0.1 mM and 1 mM. At those values, the yield was not compromised and the iodine content in leaves fitted into the recommended daily intakes established for human beings, considering the low amount of basil leaves that can enter human diet either directly as a culinary herb or after processing.

Plant nutritional value was positively affected by the iodine treatments, as an increase of leaf antioxidant power and phenolic compounds’ accumulation, especially RA and cinnamic acid, was observed in KI and KIO_3_ treated plants. Nevertheless, a substantial alteration of these nutraceuticals was obtained only with concentrations of the iodine salts between 1 and 10 mM, which in turn induced yield reduction, phytotoxicity symptoms and an excessive iodine accumulation. Administration of 1 mM KI could anyway represent a good compromise between the biofortification purpose and a moderate enrichment of antioxidant compounds in basil leaves.

The EO composition was influenced by both iodine form treatments in a dose-dependent fashion in the growth chamber set-up, with large changes in the volatiles production and even shifts in the chemotype among the samples. On the contrary, the chemotype of the open field basil was not influenced at any treatment concentration and the differences that were registered among the treatments were subtle and could not alter the overall distribution of the main EOs fractions between control and iodine-treated plants.

## Supporting information

S1 Fig Phytotoxicity symptoms detected in open field grown plants. Brown necrotic areas in the upper leaves of 10 mM KI treated plants are indicated by red arrows.
(TIF)

S2 Fig Principal component analysis (PCA) loadings plot for leaf essential oils. The complete compositions of leaf essential oils extracted from the open field and growth chamber experiments samples was used.
(TIF)
