# Correction: Effect of Iodine treatments on *Ocimum basilicum* L.: Biofortification, phenolics production and essential oil composition

**DOI:** 10.1371/journal.pone.0229016

**Published:** 2020-02-05

**Authors:** Claudia Kiferle, Roberta Ascrizzi, Marco Martinelli, Silvia Gonzali, Lorenzo Mariotti, Laura Pistelli, Guido Flamini, Pierdomenico Perata

The information for affiliations 1 and 2 is switched. The correct affiliations are as follows:

Claudia Kiferle^1^, Roberta Ascrizzi^2^, Marco Martinelli^1^, Silvia Gonzali^1^, Lorenzo Mariotti^3^, Laura Pistelli^3^, Guido Flamini^2^, Pierdomenico Perata^1^

**1** PlantLab, Institute of Life Sciences, Scuola Superiore Sant’Anna, 56127 Pisa, Italy **2** Department of Pharmacy, University of Pisa, 56126 Pisa, Italy **3** Department of Agriculture, Food and Environment, University of Pisa, 56124 Pisa, Italy
