# Supplementary figures and images for: Effect of Iodine treatments on Ocimum basilicum L.: Biofortification, phenolics production and essential oil composition
Source: PLoS One. 2019 Dec 16;14(12):e0226559. doi: 10.1371/journal.pone.0226559 (PMC6913995; doi:10.1371/journal.pone.0226559)

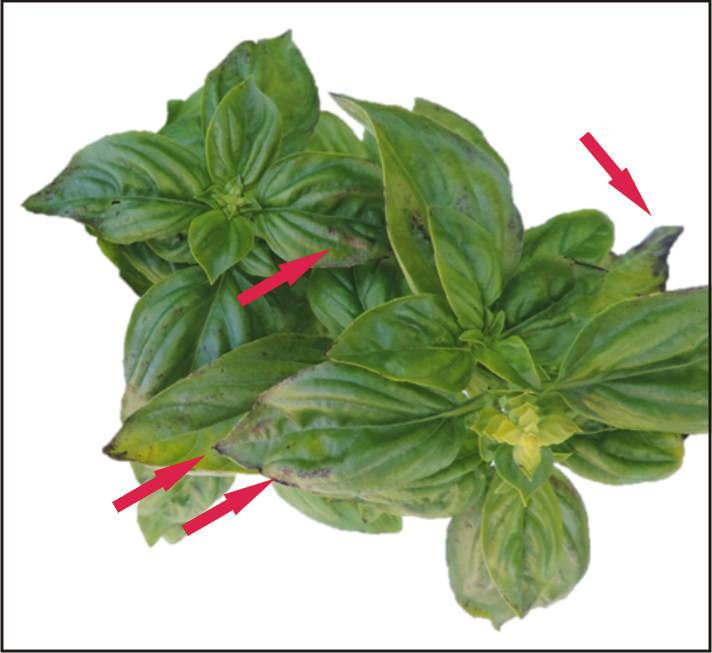

Supplement: S1 Fig — Brown necrotic areas in the upper leaves of 10 mM KI treated plants are indicated by red arrows. (TIF) [file pone.0226559.s001.tif]

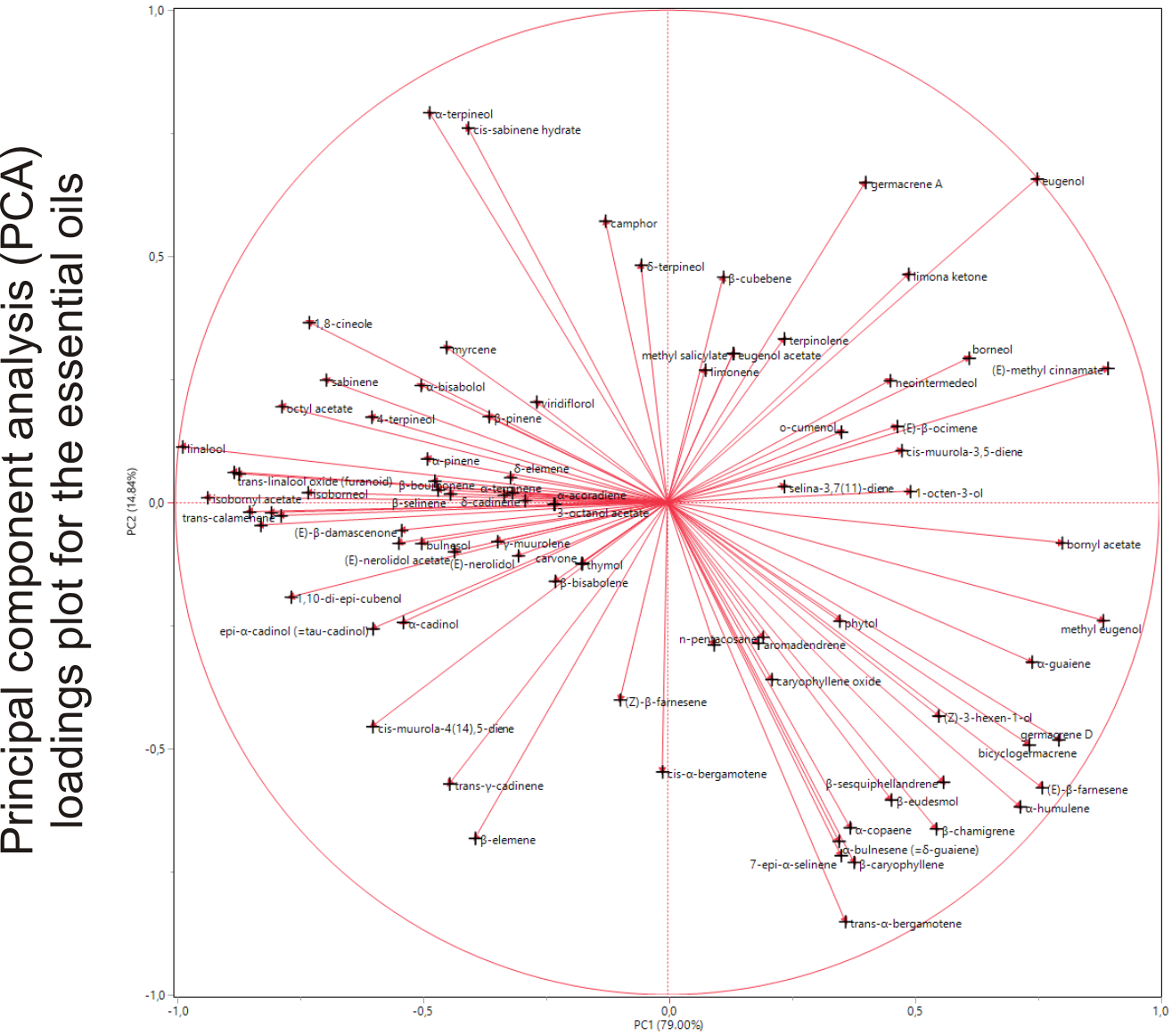

Supplement: S2 Fig — The complete compositions of leaf essential oils extracted from the open field and growth chamber experiments samples was used. (TIF) [file pone.0226559.s002.tif]
